# Hijacking of neural circuits by malignant glioma: mechanistic insights and clinical implications

**DOI:** 10.1016/j.jncc.2026.05.002

**Published:** 2026-07-03

**Authors:** Eduarda P. Martins, Helena M. Figueiredo, Nuno Sousa, Bruno M. Costa

**Affiliations:** a Life and Health Sciences Research Institute (ICVS), School of Medicine, University of Minho, Campus Gualtar, Braga, Portugal; b ICVS/3B’s - PT Government Associate Laboratory, Braga/Guimarães, Portugal; c Clinical Academic Center Braga (2CA-Braga), Braga, Portugal; d Centro Universitário Max-Planck (UniMAX), Indaiatuba, Brasil; e Centro Universitário de Jaguariúna (UniFAJ), Jaguariúna, Brasil

**Keywords:** Glioma, Cancer neuroscience, Neuronal microenvironment, Neural circuits, Hyperexcitability, Glioblastoma, Neuron-glioma synapses

## Abstract

Glioblastoma and other malignant gliomas are characterized by high heterogeneity that drives their aggressiveness and contributes to poor clinical outcomes. Understanding the mechanisms behind glioma malignancy, as well as the crosstalk between these tumors and their microenvironment, may hold transformative clinical relevance. Recent findings show that glioblastoma cells interact in bidirectional and multimodal crosstalk with the neuronal compartment of the microenvironment. While the knowledge on these complex interactions is still limited, it is now clear that dynamic communications include responses to neuronal activity-dependent molecules, synaptic signaling, and remodeling of neural circuits. Together, these direct and indirect dialogues promote glioma cells proliferation and invasion, effectively hijacking neuronal activity to fuel tumor aggressiveness. This review focuses on the bidirectional interaction between neurons and malignant glioma cells, highlighting its clinical relevance and potential to guide the development of novel precision therapies specifically targeting the glioma-neuron crosstalk.

## 1 Introduction

The molecular and cellular processes driving cancer have been extensively categorized into distinct cancer hallmarks.^1^, ^2^, ^3^ Among these, the tumor microenvironment (TME) has emerged as a critical component, profoundly influencing immune evasion, tumor-promoting inflammation, cellular metabolism, and other hallmarks of malignancy. While immune cells, epithelial cells, and fibroblasts were early recognized as key players in the TME, the contribution of the nervous system to multiple dimensions of tumor biology remained largely overlooked for many years. Recently, a growing body of evidence has highlighted the importance of neural mechanisms in the progression of multiple cancer types (elegantly reviewed by Winkler *et al.*^4^). Unsurprisingly, the emerging field of ‘cancer neuroscience’ has proven particularly compelling in the context of primary brain tumors.

Gliomas were initially conceptualized following the first description of neuroglia in 1846,^5^ which provided the cellular framework upon which these tumors would later be defined. Early histopathological observations in the late 19th and early 20th centuries established that certain brain tumors shared morphological features with glial cells, leading to their classification as "gliomas”.^6^ This histology-centric paradigm persisted for decades, relying on features such as cellular atypia, mitotic activity, microvascular proliferation, and necrosis to assign tumor classification, grade and predict patient prognosis. The identification of glioma cells has evolved substantially with molecular diagnostics. Today, gliomas are defined using integrated histological and molecular criteria, as described in the 2021 WHO Classification of Tumors of the Central Nervous System (CNS).^7^ Key molecular alterations now central to the diagnosis and classification of gliomas include *IDH*1/2 mutation status, 1p/19q codeletion, *TERT* promoter mutations, *EGFR* amplification, and histone H3 mutations. Immunohistochemistry for markers such as GFAP, IDH1 R132H, ATRX, and p53 complements molecular testing. Together, these histological and molecular tools allow the precise identification and classification of glioma subtypes. As such, gliomas comprise a diverse group of primary CNS tumors with generally poor clinical outcomes.^7^, ^8^, ^9^ Among these, glioblastoma (GBM), the most common primary malignant brain tumor in adults,^8^^,^^10^ is particularly devastating, with patients presenting a median survival of approximately 15 months after diagnosis.^11^^,^^12^ GBM’s highly infiltrative nature prevents complete surgical resection and contributes to a range of debilitating neurological symptoms, such as seizures, headaches, personality and behavioral changes, cognitive dysfunction, and memory impairments.^13^, ^14^, ^15^, ^16^ These debilitating clinical manifestations may, at least in part, reflect the extensive interactions between glioma and brain cells, although the molecular and cellular dynamics underpinning these connections have remained poorly understood.

Importantly, recent findings reveal a complex, bidirectional interaction between malignant glioma cells and neurons.^17^, ^18^, ^19^ Beyond invading the brain parenchyma, glioma cells have been shown to also disrupt neural circuits and hijack neuronal activity to further enhance their aggressiveness. These interactions include both direct mechanisms, such as *bona fide* synapses between neurons and glioma cells, and indirect effects, such as glioma-induced neuronal hyperexcitability.^17^^,^^19^^,^^20^ Collectively, this crosstalk sustains tumor aggressiveness, through enhanced tumor proliferation and invasion,^17^, ^18^, ^19^ and impairs brain function by promoting neuronal dysfunction, which can manifest clinically as neurological symptoms, such as seizures.^20^^,^^21^

In this review, we summarize and discuss foundational discoveries on these complex, multimodal, and bidirectional interactions between malignant glioma cells and neurons. We provide an overview of how glioma cells respond to neuronal activity through paracrine signaling and synaptic interactions, and how these mechanisms contribute to tumor proliferation and invasion. Furthermore, we discuss how glioma impacts neuronal activity, mainly through hyperexcitability, creating a cycle that perpetuates disease progression. These insights deepen our understanding of the molecular and cellular biology of malignant gliomas, and may help identify novel therapeutic targets. In particular, we highlight recently tested drugs aimed to modulate neuron-glioma interactions, and address the still unexplored questions and challenges that must be overcome to fully decode ‘cancer neuroscience’ in malignant gliomas. By exploring the molecular, cellular, synaptic, and electrophysiological dimensions of these interactions (Fig. 1), we provide a comprehensive framework for understanding their clinical and therapeutic implications.

**Fig. 1:**
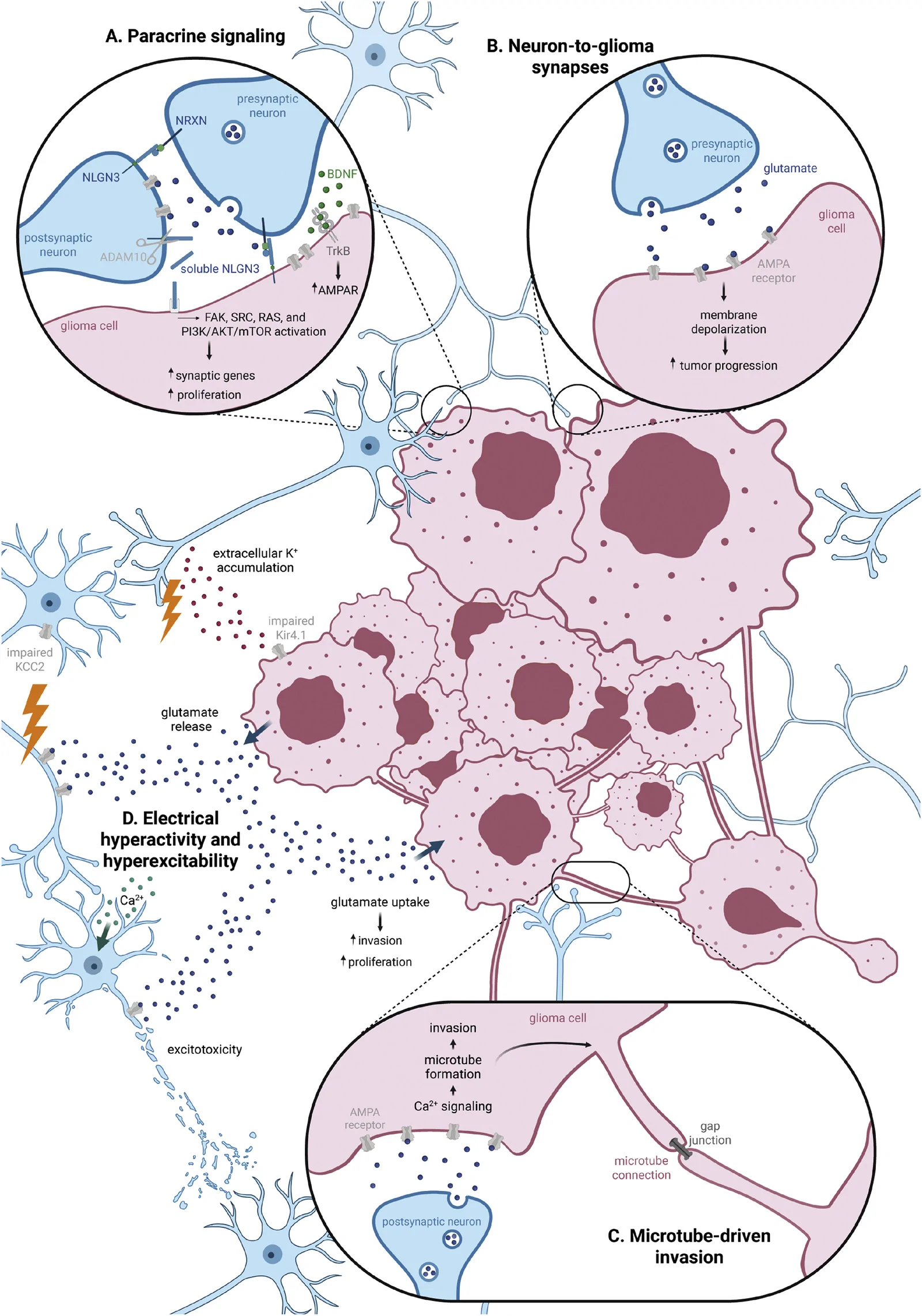
Mechanisms of neuron-glioma interactions promoting tumor progression. Glioma cells establish complex interactions with neurons that not only drive tumor growth and invasion, but also disrupt normal neuronal function, leading to network hyperexcitability and neurodegeneration. (A) Paracrine signaling: neurons release factors (e.g., NLGN3) that stimulate glioma growth and invasion. (B) Neuron-to-glioma synapses: direct synaptic connections are formed between neurons and glioma cells, allowing neuronal signals (e.g., glutamate) to enhance tumor progression and resistance to treatment. (C) Microtube-driven invasion: neuronal activity influences the microtube network in glioma cells, facilitating intercellular communication and distant invasion, while also interfering with neuronal connectivity. (D) Electrical hyperactivity and hyperexcitability: glioma-induced accumulation of extracellular glutamate and potassium (K^+^) disrupts neuronal homeostasis, leading to excessive electrical activity. This imbalance contributes to seizures while also promoting neuronal damage and creating positive feedback loops by further amplifying paracrine signaling (A) and neuron-to-glioma synaptic activity (B), ultimately driving tumor progression. Figure adapted from images created with BioRender.com.

While this review integrates findings across different glioma types, its primary focus is on GBM. This emphasis reflects both the clinical relevance of this tumor type and the fact that most mechanistic studies on neuron-glioma interactions have been conducted in GBM-derived models. Nonetheless, gliomas represent a highly heterogenous group of tumors that span pediatric and adult forms with diverse molecular and clinical features, encompass various malignant grades, and include both IDH-mutant and IDH-wildtype entities. These distinctions are accompanied by unique molecular profiles, cells of origin, and microenvironmental landscapes, which are likely to shape the bidirectional communication between neurons and malignant cells. Despite this complexity, the extent to which glioma type and anatomical context shape neuron-glioma crosstalk remains largely unexplored. These dimensions are further discussed throughout this review.

## 2 The intricacies of neuronal influence on glioma

Malignant gliomas receive a multitude of inputs from surrounding neurons that affect various cancer hallmarks, impacting tumor progression and ultimately clinical outcomes.

### 2.1 Neuronal activity as a driver of glioma growth

The discovery of the pivotal role of neuronal activity in glioma progression, highlighting a dynamic interplay between neural circuits and tumor biology that fosters pro-tumorigenic effects, has revolutionized the field. One of the most striking examples of the impact of these findings is the observation that neuronal activity promotes high-grade glioma (HGG) growth through the secretion of neurologin-3 (NLGN3), cleaved by ADAM10.^22^^,^^23^ In this context, ADAM10 promotes HGG progression by acting as a membrane-bound protease that cleaves NLGN3 and releases its soluble form into the TME, thereby enabling the activation of oncogenic pathways such as FAK, SRC, RAS, and PI3K/AKT/mTOR, inducing transcriptional changes that include the upregulation of *NLGN3* and other synapse-related genes, and proliferation of HGG cells (Fig. 1A).^22^^,^^23^ Importantly, the inhibition of ADAM10 to block NLGN3 shedding reduces HGG growth *in vivo*,^23^ demonstrating the critical relevance of this neuroligin for tumor aggressiveness. Interestingly, NLGN3 is also described as a key molecule in the initiation of optic glioma,^24^ further highlighting its relevance in gliomas.

Another activity-dependent molecule that emerged as a crucial player in glioma progression, particularly in HGG, is brain-derived neurotrophic factor (BDNF). Upon neuronal activation, BDNF is released by the synaptic membrane and binds to TrkB receptors expressed on glioma cells. This engagement triggers a set of tumor-promoting processes that act both at the synaptic and intracellular levels.^22^^,^^25^ Notably, BDNF-TrkB signaling activates MAPK-ERK, PI3K-AKT and CAMKII calcium signaling in glioma cells.^25^ Of these, CAMKII is particularly relevant in increasing the trafficking of α-amino-3-hydroxy-5-methyl-4-isoxazole propionic acid (AMPA) receptors to the malignant cell membrane, enhancing glutamate-evoked currents and strengthening neuron-to-glioma synaptic responses.^25^ These synaptic alterations amplify the level of excitatory stimulation on glioma cells and contribute to tumor plasticity.^25^ Importantly, optogenetic manipulation of glioma cells demonstrated a direct link between the magnitude of membrane depolarization and proliferative capacity, establishing that stronger depolarization inputs, mediated in part by BDNF-TrkB signaling, promote increased glioma proliferation.^25^ In addition to its effect on synaptic signaling in glioma cells, TrkB activation is known to trigger various intracellular signaling pathways with well-established tumor-promoting effects, including the PI3K-AKT and MAPK/ERK pathways.^26^^,^^27^ This intracellular signaling likely acts synergistically with the synaptic effects mediated by BDNF-TrkB, sustaining glioma progression and positioning this molecular axis as a critical mechanism through which glioma cells hijack neuronal activity to shape their behavior. In the same line, other neuronal activity-dependent released molecules are described to affect glioma through paracrine signaling: IGF-1 release by mitral and tufted neurons is associated with the initiation and progression of olfactory bulb glioma *in vivo*,^28^ and GRP78 activity-dependent secretion promotes diffuse pediatric-type high-grade glioma proliferation, albeit with a less pronounced effect compared to NLGN3.^22^ These findings underscore the critical role of neuron-derived, activity-dependent molecules in shaping the TME, promoting glioma progression and plasticity.

### 2.2 Malignant neuron-glioma synapses

Building on the discovery of NLGN3 importance for HGG, subsequent studies revealed that this neuroligin also plays a crucial role in promoting pathologic neuron-to-glioma synaptogenesis,^19^ marking a significant step forward in understanding the intricate interplay between gliomas and the neural circuits. These malignant neuron-to-glioma synapses (Fig. 1B) are electrophysiologically functional, mostly mediated by calcium (Ca^2+^)-permeable AMPA receptors (AMPAR), and present typical synaptic structures.^17^^,^^19^ Interestingly, approximately 10% of glioma cells processes were found to be involved in neuron-interacting synapses, with immune-electron microscopy confirming their localization on the postsynaptic side of synaptic structures.^19^ Glioma cells expressing AMPAR respond to excitatory signals from neurons in glutamatergic synapses,^17^^,^^19^ generating excitatory postsynaptic currents that lead to glioma cell membrane depolarization. Critically, this depolarization carries functional relevance, as it promotes glioma proliferation^19^ and GBM invasion.^18^ Importantly, radiotherapy has also been shown to enhance the connectivity between neurons and GBM cells by increasing neuronal activity.^29^ Notably, therapeutic outcomes improve when radiotherapy is combined with pharmacological inhibition of neuronal activity, emphasizing the role of neuron-to-glioma synapses in resistance mechanisms,^29^ and highlighting that neurons are not passive bystanders in the glioma TME.

Transcriptomic analyses from multiple studies revealed that glioma cells acting in malignant neuron-to-glioma synapses exhibit distinct expression profiles, enriched for oligodendrocyte precursor cells (OPC)/neural precursor (NPC)-like cell states, and neuronal and neurodevelopmental signatures.^18^^,^^19^ The OPC-like signature aligns with the observation that OPCs are frequently found as postsynaptic participants,^30^ suggesting a predisposition of glioma cells to receive neuronal signals. Distinctly, GBM cells also exhibit migration patterns resembling those of neurons, including Lévy-like movement, which may further facilitate their invasive behavior in the brain,^18^ suggesting that GBM cells present remarkable plasticity that might mimic neurons in processes beyond synaptic signaling. Additionally, GBM infiltrating cells upregulate Notch signaling, involved in synaptic plasticity,^31^ and genes related to synaptic functions.^32^ Together, these findings highlight how HGGs exploit plasticity processes impairing normal brain function and accelerating tumor progression.

Notably, the role of neuronal input in glioma stem-like cells (GSCs) remains relatively underexplored. Nonetheless, the findings described above, showing that neuron–glioma synaptic interactions and activity-dependent factors such as NLGN3 induce transcriptional programs enriched in neurodevelopmental and progenitor-like signatures,^22^ suggest a potential link between neuronal signaling and stem-like tumor states.^22^ In particular, glioma cells receiving synaptic inputs are frequently associated with OPC-like or NPC-like states, which are widely linked to stem-like properties and increased tumorigenic potential.^18^^,^^19^ Additionally, BDNF–TrkB signaling enhances synaptic plasticity and tumor cell proliferation, further supporting a putative role for neuronal activity in sustaining GSCs populations.^25^ Consistently, paracrine BDNF signaling from differentiated glioma cells has also been shown to promote glioma stem-like cell growth through NTRK2 activation, reinforcing the relevance of this pathway in stem-like tumor states.^33^

Having in consideration the identification of synapses between neurons and glioma,^18^^,^^19^ subsequent studies explored the brain location and properties of neurons that innervate gliomas. Neurons and glioma cells communicate across distant brain regions, particularly callosal projection neurons from the contralateral hemisphere that were shown to promote GBM progression and infiltration.^34^ Remarkably, the leading edge of tumors in both human samples and preclinical mouse models is enriched with an activity-dependent population of infiltrating GBM cells expressing high levels of axonal guidance genes.^34^ Among them, SEMA4F has been identified as a critical driver of activity-dependent tumor progression.^34^ An additional study demonstrated the neurons innervating glioma cells arise from distinct neuromodulatory centers and specific cortical layers, dependent on the tumor’s location.^35^ Interestingly, long range glioma-innervating neurons are predominantly glutamatergic, but some express both glutamatergic and gamma-aminobutyric acid (GABA)ergic markers, whereas local striatal glioma-innervating neurons are largely GABAergic.^35^ Additionally, electrophysiological recordings showed that glioma-innervating neurons differ from cortex-innervating neurons in non-tumor brain regions in terms of action potential properties.^35^ Recent studies have also suggested that cholinergic neurons from the basal forebrain contribute to GBM aggressiveness by promoting migration and proliferation.^29^ Consistently, stimulation of GBM organoids with acetylcholine leads to sustained increases in Ca^2+^ oscillations and enhances tumor cell migration.^36^ Critically, inhibition of the acetylcholine receptor CHRM3 reduces tumor growth and prolongs survival in GBM mouse models.^29^^,^^36^ Thus, decoding the multiple effects of cholinergic and GABAergic signaling in malignant glioma could provide important insights, complementing the relevance of glutamatergic signaling. The existence of excitatory and inhibitory neurons associated with glioma highlights the ability of these malignant cells to respond to neurotransmitters that convey opposing signals.

In addition to the capacity of glioma cells forming direct synapses with neurons, recent evidence has shown they can also indirectly influence synaptic signaling and neuronal activity by taking on astrocyte-like roles. These include perisynaptic contacts,^17^ modulation of glutamate levels,^37^, ^38^, ^39^ and regulation of synaptic plasticity,^25^ all of which are typically managed by astrocytes.^40^^,^^41^ In the context of gliomas, these actions help the tumor integrate into and reshape the brain’s neural circuits, thereby supporting tumor growth and progression.

The existence of both direct and indirect neuron-to-glioma synapses has critical implications for glioma biology. The ability of GBM cells to mimic neural properties, while integrating interconnected networks, enhances their adaptability within the brain microenvironment and cancer aggressiveness features, including therapy resistance.^18^^,^^29^^,^^35^

### 2.3 Tumor invasion and microtube formation mediated by neural activity

Beyond facilitating glioma proliferation, neuronal activity plays a pivotal role in shaping the physical architecture of the tumor by promoting the formation of long, filamentous cytoplasmic extensions, called microtubes (Fig. 1C). These allow tumor cells to form cellular networks that facilitate tumor spread, intercellular communication, and resistance to therapy.^42^ A key component of this network is gap junction-mediated coupling, which links glioma cells through the tumor microtubes, enabling direct electrical coupling and facilitating the exchange of ions and signaling molecules.^42^ This connectivity may explain how glioma cells exhibit prolonged currents with low input resistance, as gap junction blockers, such as carbenoxolone and meclofenamate, reduce the amplitude of prolonged glioma currents.^19^ Additionally, glioma cells exhibit spontaneous and stimulus-evoked synchronous Ca^2+^ transients, which can be abolished by gap junction inhibition, reinforcing the existence of a coordinated glioma network.^19^ GJB2, a gap junction molecule, and SCN9A, a voltage-gated sodium channel, were found to be significantly overexpressed in GBM and associate with worse overall survival in patients.^43^ Importantly, *GJB2* knockdown deregulates proliferative and neuron projection pathways, disrupts tunneling nanotube formation, and reduces tumor invasion *in vivo*,^43^ highlighting a critical interplay between neuronal signaling and nanotube-mediated invasion in GBM, and suggesting a potential therapeutic opportunity targeting key molecules involved in gap junctions.

Interestingly, recent evidences have uncovered new roles of neuronal activity in regulating microtube formation and promoting cell invasion in GBM. This phenomenon was demonstrated by Venkataramani *et al.*, revealing that neuron-to-glioma synapses are located on tumor microtubes and generate postsynaptic Ca^2+^ currents mediated by AMPAR.^17^ This study showed that the subpopulation of GBM cells functionally connected to neurons on tumor microtubes exhibit significantly greater invasive behavior.^17^ Moreover, episodes of tumor cell migration are closely correlated with the frequency of Ca^2+^ signals and genetic disruption of AMPAR signaling significantly reduces GBM invasion *in vivo*,^17^ clearly establishing a key mechanism amenable to therapeutic targeting.

Additionally, glioma cells interconnected through tumor microtubes exhibit synchronized rhythmic electrical activity.^44^ This rhythmic activity amplifies Ca^2+^ signaling, further promoting *de novo* formation of tumor microtubes and increasing the invasiveness of GBM cells.^18^^,^^44^ Notably, another subpopulation of GBM cells remains unconnected with other malignant cells in the infiltration zone. Curiously, these cells still engage in neuron-glioma crosstalk, selectively avoiding interactions with astrocytes, while establishing synaptic connections with neurons.^18^ Given the key role of Ca^2+^ in microtube formation and invasion, it would be important to investigate whether Ca^2+^-targeting strategies, some of which have previously been shown to decrease GBM growth,^45^^,^^46^ are able to disrupt the neuron-glioma interactions that support tumor progression.

Collectively, these studies reveal how GBM hijacks physiological neuronal activity and other microenvironmental signals to drive tumor microtubes formation, uncovering novel mechanisms that enhance invasion and establish functional tumor networks.

## 3 Neuronal hyperactivity and neurotoxicity induced by gioma

GBM profoundly alters the excitability and function of surrounding neurons, inducing hyperactivity, excitotoxicity and circuit disfunction.^19^^,^^47^^,^^48^ These complex interactions contribute to tumor progression and clinical symptoms such as seizures.

A major contributor to this dysregulation is glutamate, whose extracellular levels are aberrantly high due to glioma-mediated overexpression of system $X_{c}^{-}$, a cystine/glutamate antiporter, which exports glutamate in exchange for cystine uptake.^49^ This mechanism supports tumor growth and oxidative stress resistance, while also leading to excessive release of glutamate in the peritumoral environment (Fig. 1D).

Elevated extracellular glutamate concentrations promote neuronal hyperexcitability by overactivating glutamate receptors, such as N-methyl-D-aspartate (NMDA) and AMPA receptors, on neuronal membranes.^38^ This excessive activation depolarizes neurons, and enhances synchronized neuronal firing by lowering the threshold for action potential generation, ultimately leading to hyperexcitability.^38^ Importantly, some pharmacological strategies have explored this hyperexcitability phenomenon in malignant gliomas. For example, sulfasalazine inhibits system $X_{c}^{-}$-mediated glutamate release from gliomas, reducing peritumoral hyperexcitability and epileptic events in GBM-bearing mice.^38^ Moreover, perampanel, an AMPAR antagonist, has been investigated in a small cohort of HGG patients to assess its efficacy in reducing seizure frequency. This pilot trial demonstrated that this drug effectively reduces the proportion of patients experiencing seizures, highlighting its potential to alleviate one of the most debilitating glioma-related symptoms.^50^ However, no significant improvement was observed in patient overall survival or in the primary hyperexcitability parameter evaluated.^50^ Together with limited number of patients included in the trial, these findings underscore the need for further research to fully understand the therapeutic potential of perampanel. Encouragingly, a phase II trial specifically targeting GBM has been initiated to test its clinical potential to reduce tumor cell network connectivity and tumor growth.^51^ Beyond its role in hyperexcitability and seizures, elevated release of glutamate by glioma cells^37^, ^38^, ^39^ leads to an overactivation of NMDA and AMPA receptors and subsequent Ca^2+^ influx, promoting neuronal excitotoxicity, cell death and affecting normal brain function.^47^^,^^48^

In addition to glutamate-mediated hyperexcitability and excitotoxicity, GBM cells disrupt GABAergic signaling by impairing the neuron-specific K^+^-Cl^–^ cotransporter 2 (KCC2), which normally maintains low intracellular Cl⁻ levels by actively exporting it from the neuron. When KCC2 is compromised, intracellular Cl⁻ accumulates, inverting the Cl⁻ electrochemical gradient.^52^^,^^53^ As a consequence, GABA receptor activation leads to Cl⁻ efflux rather than influx, depolarizing rather than hyperpolarizing the neuron, and thus reversing GABA's effects from inhibitory to excitatory.^52^^,^^53^ Notably, BDNF-TrkB signaling, previously discussed as a promoter of glioma proliferation and synaptic plasticity (Section 2.1), has also been identified as a key regulator of KCC2 activity, suggesting that this pathway may simultaneously drive tumor progression and contribute to neuronal hyperexcitability through KCC2 impairment.^52^ These combined effects further amplify hyperexcitability by increasing excitatory signaling, and promote epileptic activity.^53^

The normal neuronal excitability is also regulated by potassium (K^+^) ions, which help restore the membrane potential during repolarization following an action potential.^54^ In GBM, the ability to spatially buffer extracellular K^+^ through K^+^ selective channels, such as Kir4.1, is impaired, increasing extracellular K^+^ levels.^20^^,^^21^ Additionally, recent studies have revealed that OPC-like cells within the TME also contribute to K^+^ dysregulation.^55^ These cells, enriched at the cancer-neuron interface in epileptic GBM patients, express the voltage-gated K^+^ channel KV4.2.^55^ Dysregulated KV4.2 activity in OPC-like cells further promotes neuronal hyperexcitability by enhancing extracellular K^+^ accumulation.^55^ Elevated extracellular K^+^ reduces the driving force for its efflux, impairing neuronal repolarization. This sustained depolarization increases the likelihood of synchronized neuronal firing and seizure generation.

Additionally, GBM-derived extracellular vesicles (EVs) can also induce neuronal synchronization or desynchronization depending on the state of the neuronal network.^56^^,^^57^ The induction of synchrony of immature neurons is caused by increased connectivity, originating from a higher number of synaptic contacts.^56^ On the other hand, desynchronization of mature neurons following GBM-EVs exposure is driven by an imbalance between increased excitatory and decreased inhibitory synaptic inputs.^56^ Moreover, GBM-derived EVs from both plasma and tumor cell lines have been shown to induce IgG-mediated, complement-dependent apoptosis and necrosis of neurons, further contributing to glioma-induced neurotoxicity.^58^

Glioma cells were also recently found to cause loss of dendritic spines and impair inhibitory synapses via mTOR activation of neighboring neurons.^59^ Importantly, inhibiting mTOR with AZD8055 reverses these effects, reducing excessive excitatory activity, restoring dendritic spine density, and normalizing synaptic transmission, ultimately mitigating glioma-induced neurological dysfunction.^59^

While excessive glutamate negatively affects neurons, it also serves as an autocrine pro-tumoral signal to glioma cells. By activating AMPARs and NMDARs, and mediating intracellular Ca^2+^ oscillations, glutamate enhances glioma cell migration and tumor growth.^17^^,^^37^^,^^60^, ^61^, ^62^ In addition to the effects on neurons and glioma cells, glutamate also modulates immune cells, suppressing mast cells^63^ and reducing CD8^+^ T cells responses,^64^ constituting another mechanism by which this neurotransmitter-mediated crosstalk shapes the TME. Moreover, neuronal hyperactivity itself is not just a consequence of glioma presence, but also actively promotes tumor progression. Increased neuronal activity in the peritumoral environment drives the release of key neurotrophic factors, such as BDNF, which GBM cells exploit to enhance their proliferation and survival.^19^^,^^25^

Overall, the interplay between glioma cells and neural circuits creates a self-reinforcing loop in which dysregulated glutamate signaling, impaired ion homeostasis, and tumor-derived EVs exacerbate neuronal hyperactivity, excitotoxicity, and synaptic dysfunction. Targeting these pathways, particularly through therapies like sulfasalazine and perampanel, may offer a promising avenue to reduce neuronal hyperactivity, alleviate patient symptoms, and potentially slow glioblastoma progression.

Collectively, the mechanisms of bidirectional interaction between glioma cells and neurons, ranging from paracrine signaling and functional synapses to microtube-driven invasion, converge into a self-amplifying feedback loop that drives disease progression (Fig. 2). At its core, glioma cells release excessive glutamate via the system $X_{c}^{-}$, inducing neuronal hyperexcitability.^38^^,^^49^ This heightened neuronal activity stimulates the release of pro-tumorigenic factors from hyperexcitable neurons, including NLGN3 and BDNF,^22^^,^^23^^,^^25^ which in turn activate oncogenic signaling pathways in glioma cells, promoting proliferation and invasion. BDNF-TrkB signaling further amplifies this loop by upregulating AMPAR trafficking to the glioma cell membrane,^25^ thereby enhancing synaptic responses and membrane depolarization that drive proliferation. Simultaneously, glioma-induced impairment of KCC2 and Kir4.1-mediated ion buffering further elevates neuronal excitability, sustaining the release of pro-tumorigenic neuronal signals.^20^^,^^21^^,^^53^ At the structural level, Ca²⁺ oscillations driven by synaptic inputs promote the formation of tumor microtubes and gap junction-coupled glioma networks.^17^^,^^44^ These networks facilitate invasion and increase the surface area available for new neuron-glioma synaptic contacts, perpetuating the bidirectional cycle. Additionally, GBM-derived EVs further modulate neuronal activity, adding another layer of bidirectional communication that feeds back into tumor progression.^56^^,^^57^ Notably, even therapeutic interventions like radiotherapy may inadvertently reinforce this loop by enhancing neuronal activity and neuron-tumor connectivity,^29^ highlighting the need to combine standard treatments with strategies targeting neuron-glioma interactions. As the tumor grows and invades, glutamate release is further amplified, escalating the entire cycle. Importantly, this framework suggests direct clinical implications: disrupting any single node - such as targeting glutamate release with sulfasalazine,^38^ blocking AMPAR-mediated signaling with perampanel,^50^^,^^51^ inhibiting NLGN3 shedding via ADAM10 inhibitors,^23^ or disrupting gap junction coupling with meclofenamate^19^ - could break the cycle and slow tumor progression. This perspective underscores the importance of viewing neuron-glioma interactions not as isolated events but as a dynamic, mutually reinforcing process that is central to glioma pathology and that may be most effectively targeted through combinatorial therapeutic strategies.

**Fig. 2:**
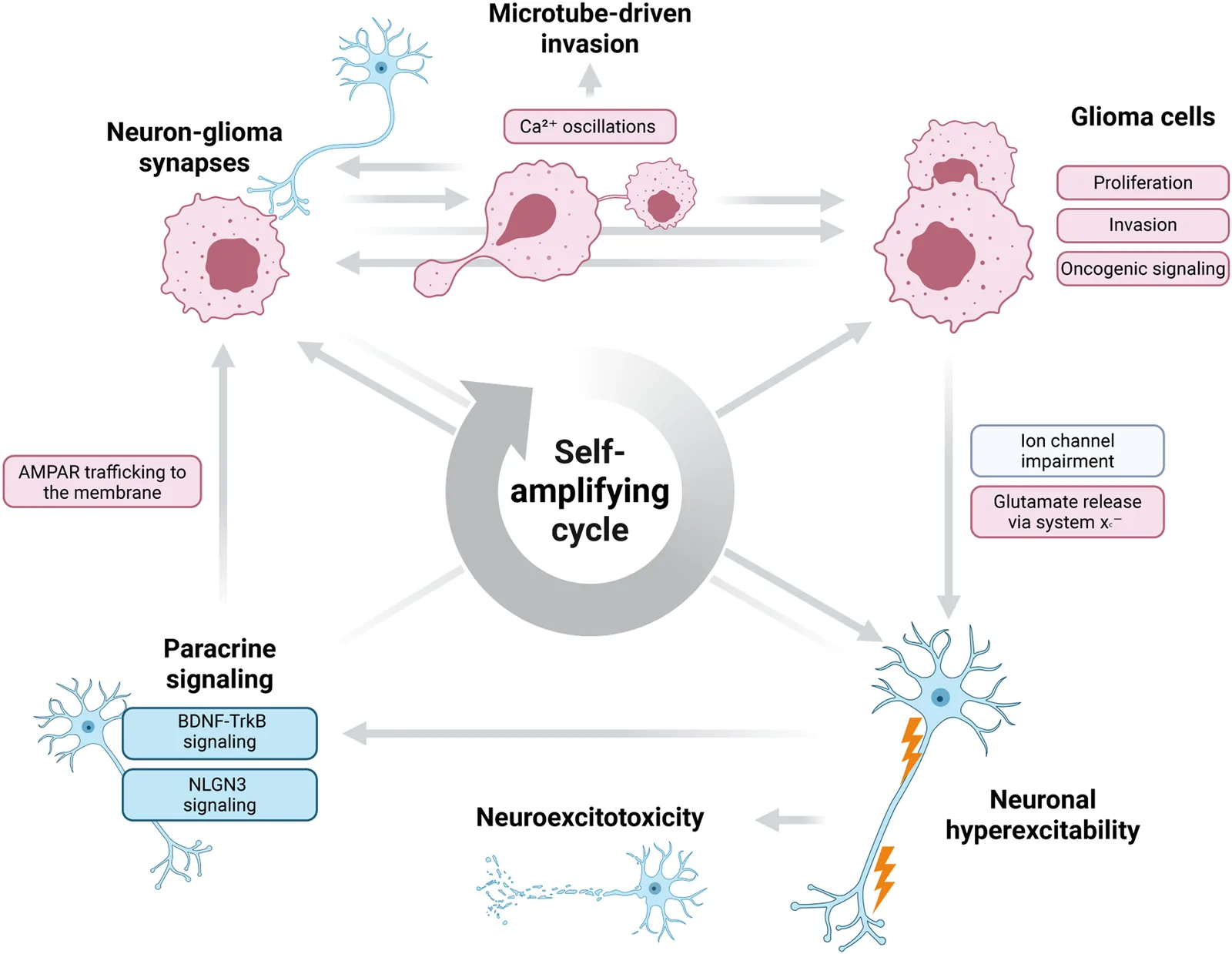
Integrated neuron–glioma self-amplifying feedback loop driving tumor progression. Glioma cells release excessive glutamate via the system $X_{c}^{-}$, inducing neuronal hyperexcitability. In turn, hyperexcitable neurons secrete pro-tumorigenic factors, including NLGN3 and BDNF, which activate oncogenic signaling pathways in glioma cells to drive proliferation and invasion. BDNF–TrkB signaling amplifies this process by upregulating AMPAR trafficking to the glioma cell membrane, thereby strengthening synaptic responses and membrane depolarization. Concurrently, impaired neuronal ion buffering due to ion channel deregulation elevates neuronal excitability and sustains the release of tumor-promoting signals. Chronic hyperexcitability may ultimately lead to neuronal excitotoxicity, further facilitating tumor expansion, further facilitating tumor expansion. Synaptically-driven Ca²⁺ oscillations promote the formation of tumor microtubes and gap junction–coupled glioma networks, facilitating invasion and increasing neuron–glioma connectivity. Collectively, these interconnected mechanisms form a self-reinforcing, non-linear network that perpetuates glioma progression. Figure adapted from images created with BioRender.com.

## 4 Therapeutic opportunities at the glioma-neuron crosstalk

GBM and other malignant gliomas remain highly aggressive and lethal brain tumors. Importantly, it is now known that they pose the ability to hijack normal brain cells to enhance their pro-tumoral features. This capacity is fundamental for their plasticity and underscores their ability to exploit the neural microenvironment to maintain malignant progression.

Therefore, as these mechanisms impact the clinical outcome of the patient, it is critical to focus on targeting neuron-tumor interactions for future therapeutic intervention. Strategies to interfere with the communication between neurons and glioma cells could be beneficial, offering a way to dissociate the tumor from the host brain and potentially improve patient clinical outcome. As discussed throughout this review, various drugs are being explored and showing promising results in affecting these interactions between neurons and glioma cells (Table 1), such as emerging neuromodulatory drugs, including AMPAR inhibitors (e.g., perampanel),^17^^,^^50^^,^^51^ and gap junctions blockers (e.g., carbenoxolone and meclofenamate).^19^ Importantly, targeting paracrine signaling mechanisms has also shown promise: inhibitors of ADAM10 have been demonstrated to reduce NLGN3 secretion and tumor growth in HGG models.^23^ Moreover, the use of TrkB inhibitors effectively disrupts BDNF-mediated signaling, decreasing proliferation and neuron-to-glioma synapse formation in diffuse midline glioma.^25^ Additionally, cellular reprogramming is also arising as a possible strategy to tackle these associations. Some examples include the overexpression of the transcription factor neuronal differentiation 4 (NeuroD4) that reprograms GBM cells into a neuron-like phenotype, through the SLC7A11-GSH-GPX4 signaling cascade;^65^ or the exogenous expression of NRXN1 in GBM that shifts the differentiation toward a neuronal-like phenotype reducing tumor aggressiveness.^66^

**Table 1:** Promising drugs under investigation with potential to target key cancer neuroscience mechanisms in malignant glioma.

Main targeted mechanism	Drug	Mechanism/target	Cancer type	Representative studies with pre-clinical findings and clinical trials
Paracrine signaling targeting	INCB7839 (aderbasib)	ADAM10 and ADAM17 inhibitor	HGG	*In vivo*: Reduced tumor growth of orthotopic pediatric GBM tumors.^23^ Clinical trials: Phase 1 (NCT04295759) in children with recurrent/progressive HGG – completed, demonstrating that the inhibitor is generally well tolerated.

	GI254023X	ADAM10 inhibitor	DIPG/HGG	*In vitro*: GI254023X reduced the secretion of NLGN3 from glioma cells primed with NLGN3.^23^ GI254023X delayed the integration of GSCs spheres into organoids, where GSCs can interact with neurons and form structures similar to hemisynapses.^88^ *In vivo*: Reduced tumor growth and proliferation index of orthotopic pediatric GBM and DIPG tumors.^23^ GBM-bearing brain slices treated with GI254023X showed decreased NLGN3 secretion.^23^ Treatment with nanoparticles encapsulated with GI254023X, CuS and RuBi-GABA prolonged the survival of orthotopic glioma mouse models.^89^

Entrectinib	TrkB inhibitor	DIPG/HGG	*In vitro*: Decreased number of neuron-to-glioma synaptic structures in neuron-glioma co-cultures.^25^ Entrectinib abrogates BDNF effect in the proliferation of DIPG.^25^ *In vivo*: Decreased tumor proliferation index and prolonged survival of patient-derived pediatric DIPG orthotopic mice model.^25^ Clinical trials: Phase 2 (NCT06528691) in < 3 years old patients with NTRK1/2/3- or ROS1-fused HGG – recruiting. Phase 2/3 (NCT05770544) in pediatric and young adults (various cancer types considered eligible) – recruiting. Phase 1/2 (NCT02650401) in pediatric patients with solid tumors or primary CNS tumors – active without posted results.

Larotrectinib	TrkB inhibitor	DIPG/HGG	*In vitro*: Larotrectinib abrogates BDNF effect in the proliferation of DIPG.^25^ Clinical trials: Early phase 1 (NCT04655404) in children with HGG with TRK fusion – recruiting. Phase 2 (NCT03213704) in patients with non-Hodgkin lymphoma, solid tumors, including recurrent glioma, or histiocytic disorders with NTRK fusions – active without posted results. Phase 2 (NCT02465060) in adult patients with lymphoma, multiple myeloma or solid tumors including glioma – active without posted results.

Targeting neuron-glioma synapses	Haloperidol	DRD2 inhibitor	GBM	*In vitro*: The DRD2 expression level inversely correlates with TMZ sensitivity in MGMT-deficient GBM.^90^ *In vivo*: Haloperidol induces ferroptosis and effectively enhances TMZ efficacy. Mechanistically, haloperidol counteracts TMZ-induced cAMP elevation, which activates PKA and triggers ER stress–mediated autophagy.^90^

	SKF83566	DRD1 inhibitor	GBM	*In vitro*: DRD1 contributes to GBM invasion and progression by regulating c-Myc entry into the nucleus that affects the transcription of the UHRF1 gene. *In vivo*: SKF83566 inhibits the transmembrane protein DRD1.^91^

Perampanel	AMPA receptor antagonist	Glioma	*In vivo:* Administration of perampanel to xenografted mice decreased the proliferation of GBM cells and invasion and growth were also reduced by anaesthesia and the AMPA receptor antagonist perampanel.^17^ Clinical trials: A phase 1/2 trial (NCT04497142) demonstrated that this drug reduces the proportion of patients experiencing seizures in HGG patients.^50^ Phase 4 (NCT04650204) in patients with high-grade glioma and focal epilepsy – terminated with results posted. Phase 1/2 trial (NCT04497142) in patients with high-grade glioma demonstrated that treatment with perampanel did not have clinically meaningful effects on peritumoral hyperexcitability or survival.^50^ Phase 2 (EudraCT 2023-503938-52-00) in GBM patients – active without posted results.^51^
	AZD8055	mTORC1/2 inhibitor	Glioma	*In vivo*: Oral gavage administration of AZD8055 reduced epileptiform activity (seizures/interictal discharges) in glioma-bearing mice.^92^ Treatment with AZD8055 decreased the phosphorylation of ribosomal protein S6 in HA+-neurons associated with the tumor.^59^ Treatment with AZD8055 recovered the number of GPHN^+^ puncta localized to GFP^+^ neurons, which is decreased in tumor-bearing brains comparing to control brains, indicating a decrease in inhibitory synapses.^59^ Two-photon imaging revealed that AZD8055 treatment reduced the calcium peak rates in high-tumor-burdened neurons, considerably amplified when comparing tumor-burdened neurons to no-tumor neurons.^59^ Brain slices from mice treated with a single dose of AZD8055 and bathed in the inhibitor demonstrated decreased excitability.^92^ Single oral administration of AZD8055 was sufficient to partially restore the dendritic spine density, evaluated in brain slices when compared to no-tumor brains.^59^ Clinical trials: Phase 1 (NCT01316809) in patients with recurrent malignant gliomas, including GBM, gliosarcoma, anaplastic astrocytoma, anaplastic oligodendroglioma, anaplastic mixed oligoastrocytoma and malignant glioma/astrocytoma NOS – completed without posted results.

Invasion and microtube formation targeting	Carbenoxolone	Gap junction blocker	DIPG	*In vivo*: Reduced the amplitude of prolonged glioma currents while simultaneously increasing glioma input resistance.^19^

Meclofenamate	Gap junction blocker	DIPG GBM	*In vivo*: Reduced the amplitude of prolonged glioma currents while simultaneously increasing glioma input resistance.^19^ Clinical trials: Phase 1/2 (EudraCT 2021-000708-39) in MGMT-methylated GBM patients – completed without posted results.^93^

Targeting hyperactivity and hyperexcita-bility	Sulfasalazine	System $X_{c}^{-}$ inhibitor, a cystine/ glutamate antiporter	Glioma	*In vitro*: Mechanistically sulfasalazine inhibits system $X_{c}^{-}$ and induces ferroptotic cell death in glioma cells. Neurons and brain tissue are almost non-responding to sulfasalazine whereas isolated astrocytes are less sensitive towards sulfasalazine toxicity compared to gliomas.^94^ *In vivo*: Sulfasalazine treatment does not affect experimental tumor growth and treated animals revealed comparable tumor volume as untreated controls. However, sulfasalazine treatment resulted in reduced glioma-derived edema and, hence, total tumor volume burden as revealed by T2-weighted magnetic resonance imaging.^94^ Sulfasalazine decreases epileptic activity in mice.^38^ Clinical trials: Phase 2 (NCT04205357) in patients with advanced solid tumors, including glioma – completed without posted results.


Abbreviations: BDNF, brain-derived neurotrophic factor; CNS, central nervous system; DIPG, diffuse intrinsic pontine glioma; DRD, dopamine receptor; ER, endoplasmic reticulum; GBM, glioblastoma; HGG, high-grade glioma; NOS, not otherwise specified; TMZ, temozolomide.

Overall, the clinical evidence available to date remains preliminary. For some approaches, such as ADAM10/17 inhibition with INCB007839, reported data primarily relate to safety and tolerability rather than clear anti-tumor efficacy. In the case of perampanel, early clinical findings suggest a potential benefit in seizure control, but clinically meaningful improvements in peritumoral hyperexcitability or survival have yet to be demonstrated. For several other strategies listed in Table 1, clinical studies are ongoing or have been completed without sufficiently detailed efficacy results being publicly available. These observations highlight both the therapeutic promise of targeting cancer neuroscience mechanisms and the imperative for further clinical validation.

## 5 Clinical implications and future challenges

Comprehending and measuring the functional interplay between neurons and glioma cells remains a major challenge. Experimental methodologies currently available include electrophysiology, optogenetics, calcium imaging, and patch-clamp,^67^ and allow the direct quantification of synaptic signaling, neural network remodeling, and tumor responses. Despite notable advances, several challenges remain before the full potential of cancer neuroscience can be translated into clinical settings (Fig. 3). A significant barrier is the need for preclinical models that authentically capture the complex and dynamic bidirectional interactions between glioma cells and the brain. Traditional *in vitro* systems and murine models often lack functional innervation and do not reflect the structural and cellular heterogeneity of the human brain. Novel models incorporating induced pluripotent stem cells (iPSC)-derived neural components into 3D organoids, genetically engineered mouse models with integrated neural circuits, optogenetics, rabies virus-based neuronal ablation and designer receptors exclusively activated by designer drugs (DREADDs) might offer promising avenues, but broader implementation and standardization are needed. The combination of some of these strategies might help solve the fundamental mechanistic questions on additional interactions between neurons and glioma cells, the precise molecules involved in those connections, and the overall impact on the tumor and brain physiology. Furthermore, these models can better inform on the potential therapeutic benefit of some of the therapies discussed above. However, despite encouraging preclinical results, the translation of these approaches into clinical benefit remains limited. This gap between preclinical findings and clinical efficacy may be explained by several factors, including limitations of experimental models that fail to fully recapitulate the complexity of the human brain, particularly the presence of functional neuronal circuits and the structural heterogeneity of the tumor microenvironment. In addition, the high intratumoral and interpatient heterogeneity of gliomas, particularly GBM, may limit the efficacy of approaches targeting specific signaling pathways, as the adaptive plasticity of glioma cells may enable compensatory mechanisms that reduce the long-term efficacy of targeted interventions. Moreover, the blood–brain barrier poses a major obstacle to effective drug delivery and therapeutic strategies aimed at modulating neuronal activity raise concerns regarding off-target effects and neurotoxicity, given the essential role of these pathways in normal brain function. Moving forward, addressing these challenges will require the development of more relevant models that efficiently mimic tumor microenvironment, heterogeneity and plasticity, allowing an integrative analysis that better bridges the gap between the mechanistic findings obtained in preclinical models and clinical application.

**Fig. 3:**
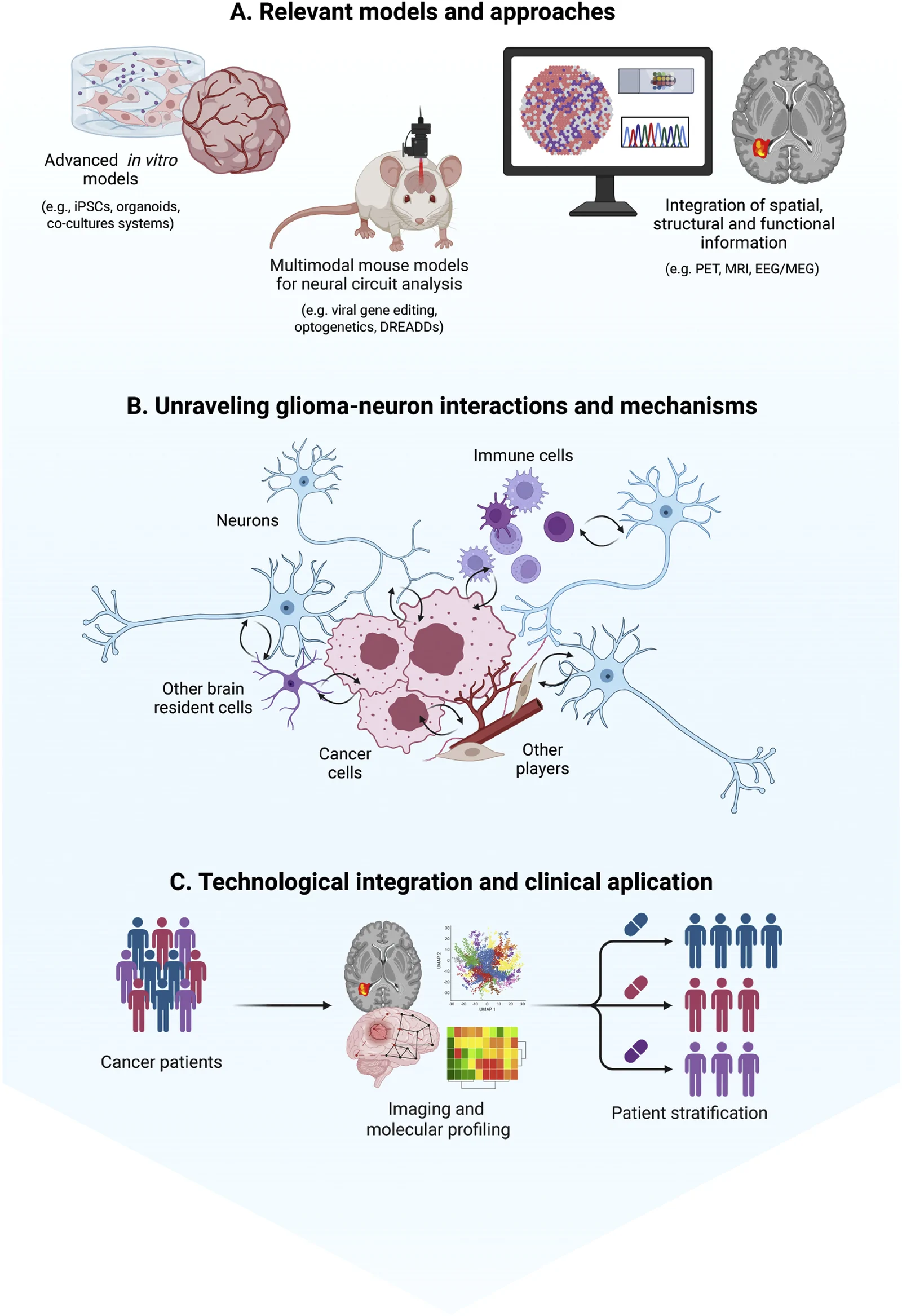
Future challenges and opportunities in the emerging field of cancer neuroscience in malignant gliomas. Holistic overview of the major challenges in deciphering the complex glioma-brain crosstalk, and translating this knowledge clinical benefit. (A) Relevant models and approaches: advancing the field requires integration of complex *in vitro* models (e.g., iPSCs, organoids, and multicellular co-cultures systems) with multimodal mouse models for broad neural circuit-level studies (e.g., gene editing of neurons via viral manipulation, optogenetic tools, DREADDs), and integration of spatial, structural, and functional information (e.g., PET, MRI, EEG/MEG). (B) Unraveling glioma-neuron interactions and mechanisms: the application of these models will further explore the known neuron-glioma interactions, and reveal new mechanisms of communication with the variety of brain-resident cells. (C) Technological integration and clinical application: the integration of the mechanistic knowledge with spatially-resolved molecular profiling and advanced imaging at the clinical setting could hold significant relevance for patient stratification and guide novel precision therapies. DREADDs, designer receptors exclusively activated by designer drugs; EEG, electroencephalography; iPSCs, induced pluripotent stem cells; MEG, magnetoencephalography; MRI, magnetic resonance imaging; PET, positron emission tomography. Figure adapted from images created with BioRender.com.

Equally pressing is the need to integrate functional and spatial technologies, such as connectomics, spatial transcriptomics and radiomics, to decode glioma-induced brain remodeling. Intriguingly, the putative associations between brain connectivity patterns and patient survival remains elusive, as recently reviewed by Salvalaggio *et al.*^68^ While some studies suggest borderline or no significant relationship between the connectome and survival in patients with malignant glioma,^69^^,^^70^ others report that higher connectivity associates with longer survival.^71^^,^^72^ Additionally, there are also evidences supporting that higher functional connectivity associates with reduced survival.^73^^,^^74^ Globally, these apparently contradicting findings may support a lack of a clear prognostic value of connectivity states in glioma patients. Nonetheless, they might also be related to methodological variability across studies, including differences in sampling strategies, connectivity metrics (functional connectivity *vs.* structural connectivity), and imaging approaches (e.g., functional magnetic resonance imaging [fMRI], diffusion tensor imaging [DTI], electroencephalography [EEG]/ magnetoencephalography [MEG]). In addition, these discrepancies may also reflect distinct biological contexts; for example, increased connectivity may, in some cases, indicate preserved or compensatory network function, which could associate with improved clinical outcomes.^71^^,^^72^ Conversely, higher connectivity may also reflect enhanced integration of glioma cells into functional neural circuits, facilitating activity-dependent tumor growth and invasion, and thereby associating with worse prognosis.^73^^,^^74^ These opposing interpretations highlight the need to distinguish between functional network preservation and tumor-driven circuit remodeling when evaluating connectome-based metrics in glioma patients.^68^ In this context, there is a clear need to develop consensus-based standards to improve the rigor, transparency, and reproducibility of connectomics research in glioma, alongside coordinated efforts to optimize sampling strategies, imaging protocols, connectivity metrics, and integrative computational models. This will be critical to establish more robust and clinically relevant connectome-based biomarkers in glioma patients.

Additionally, connectome-based parameters have demonstrated potential for predicting cognitive performance in glioma.^73^^,^^75^ These results underscore the opportunity to improve patient care by integrating connectome monitoring through PET, MRI, and EEG/MEG approaches into clinical practice, allowing a better assessment of the tumor impact on brain normal functions. Innovative technologies, such as spatially-resolved transcriptomics, can help integrate multidimensional data on tumor-brain interactions. A recent study described an increased expression of genes involved in neurodevelopmental pathways, synaptic signaling, and glial cell differentiation in GBM cells localized in infiltrated brain regions. In contrast, proneural and mesenchymal tumor cores presented differences in their transcriptomes. Interestingly, these differences are particularly prominent in the tumor core, as compared to infiltrated tissue.^32^ Furthermore, spatial profiling of both diffuse midline glioma and GBM have revealed that OPCs are enriched in the tumor core, but invasive regions, which present a high content of neurons, present an enrichment on radial glial stem-like cells.^76^ These findings highlight distinct cellular niches within individual tumors, which might contribute to distinct interactions with the normal brain.

In addition to the experimental evidence discussed here, recent theoretical and computational developments might also help decipher the interplay between neuronal activity and glioma progression. Recent studies highlight the importance of network-based and computational methods, demonstrating that the remodeling of neural circuits induced by glioma is associated with tumor progression and patient survival,^73^ and that connectome-based models can be used to predict functional outcomes such as cognitive performance in glioma patients.^75^ These models might be used to complement experimental findings by enabling the simulation of spatial and temporal patterns of tumor growth and invasion, as well as by exploring how activity-dependent changes in neural networks influence glioma behavior.

From a mechanistic perspective, glioma-induced neuronal hyperexcitability reflects altered neural network dynamics, where ionic imbalances bring neurons closer to the firing threshold. This bioelectric remodeling supports activity-dependent signaling that fosters tumor growth and invasion. In this context, theoretical and computational approaches based in dynamical systems and network models pose an invaluable methodology to better describe these interactions.^77^ As such, the integration of computational modelling and theoretical approaches with electrophysiology, advanced imaging, and connectomics data through a multidisciplinary perspective will be essential to better comprehend the whole dimension of neuron-glioma crosstalk. Supporting this view, recent integrative studies that combine molecular profiling with neural and clinical data have detected signatures with prognostic value, highlighting the translational value of this integrative approach.^74^

An additional dimension that warrants future investigation is whether neuron-glioma communication operates differently depending on the spatial scale at which it occurs. Long-range synaptic inputs from distant neurons generate rapid excitatory responses in glioma cells, mediated by AMPAR activation,^17^^,^^19^ while local microtube-coupled glioma networks exhibit slower, rhythmic electrical activity driven by gap junction coupling^42^ and Ca²⁺ oscillations.^44^ Paracrine signals, such as NLGN3 and BDNF, act over extended timescales, modulating tumor behavior on the order of minutes to hours.^22^^,^^25^ Whether and how these distinct modes of communication converge to collectively shape tumor progression remains unknown; fully unraveling these dynamics will likely require integrating electrophysiology, calcium imaging, and computational modelling.

Another promising avenue lies in the study of EVs, which are gaining recognition as mediators of tumor-neuron communication. GBM-derived EVs have been shown to influence the behavior of brain-resident cells, including neural stem and progenitor cells. These vesicles may also alter neuronal activity by modulating ion channels and synaptic firing patterns.^56^ Investigating the bidirectional nature of this communication, including the role of neuron-derived EVs, could offer valuable insights into mechanisms that enhance tumor aggressiveness. Curiously, synaptic glutamatergic activity has been shown to regulate EV release by neurons.^78^ Thus, the altered glutamate levels in the presence of the GBM tumor may disrupt the release of neuron-derived EVs, creating feedback loops that further impact tumor growth and progression. This reciprocal and dynamic communication between neurons and glioma cells is crucial, as EVs from neurons are known to carry bioactive molecules in other brain pathologies, such as Alzheimer's disease.^79^^,^^80^ Investigating whether similar mechanisms exist in the context of GBM could provide invaluable information. Additionally, EVs may also play a significant role in the complex crosstalk with the immune system. For example, neuron-derived EVs containing miR-200c-3p activate microglia signaling via the ERK pathway, promoting a M2-like phenotype that fosters GBM progression.^81^ These findings suggest that targeting EV-mediated communication could open new therapeutic avenues to disrupt the tumor’s relationship with the brain and the immune system.

While the precise mechanisms of interaction between GBM and neurons are being explored, another strategy that could hold clinical importance, helping inform on patient outcome is the identification of tumor signatures associated with tumor-brain interactions. On this basis, a recent study identified an epigenetically neural signature in GBM patients that predicts overall and progression-free survivals.^74^ High-neural GBM tumors present hypomethylated CpG sites and an increased expression of genes linked to synaptic integration, supporting their ability to foster neuron-to-glioma synapses.^74^ Specifically, both *in vivo* and *in vitro* analyses showed a higher engagement of malignant cells in neuron-to-glioma synapses in high-neural tumors as compared to low-neural GBMs. Clinically, high-neural tumors are associated with shorter overall and progression-free survivals of GBM patients, paired with increased functional connectivity, further reinforcing the link between synaptic integration and poor patient outcomes.^74^ Importantly, this neural epigenetic signature extends its relevance to diffuse midline gliomas, suggesting a common mechanism across different glioma entities.^74^ This study highlights the relevance of identifying molecular signatures that will inform on the neuron-glioma interactions and help stratify the patients.

An equally important and still underexplored dimension is how glioma types, malignancy grades, and anatomical contexts may influence neuron-glioma interactions. Emerging evidence suggests that these aspects may have direct consequences on how tumors engage with neural circuits. For instance, a recent study comparing synaptic activity across different malignancy grades of gliomas demonstrated that HGG hijack neural circuits differently from lower-grade gliomas (LGG). Neurons in cortical regions infiltrated by HGG tumors exhibit increased excitability compared to those associated with LGG, while malignant glioma cells in HGG display synaptic responses with smaller amplitudes but prolonged duration.^82^ Complementing these malignancy grade-associated differences, another study identified anatomical context-dependent variations in GABAergic signaling by comparing hemispheric GBM with diffuse midline gliomas, such as *H3K27M*-altered diffuse intrinsic pontine glioma. Notably, diffuse midline gliomas respond to GABAergic input with membrane depolarization that promotes tumor growth *in vivo*, whereas GBM cells exhibit only minimal depolarization in response to similar stimuli.^83^ Besides highlighting subtype-specific differences, this study also suggests a potential influence of anatomical context, as these tumor types arise in distinct brain regions with unique microenvironmental and neural circuit properties. Altogether, these findings support the hypothesis that bidirectional interactions between neurons and glioma cells are not constant across different tumor contexts, but are dynamically shaped by intrinsic tumor and microenvironmental features. Comprehending how these factors affect the neuron-glioma interplay will be critical to identify actionable mechanisms that might be leveraged therapeutically.

Beyond tumor-driven mechanisms of neuron-glioma interaction, an important question is whether pre-existing neurological or psychiatric conditions may influence glioma initiation or progression. Recent studies addressing this hypothesis have not identified a consistent causal effect of major neurodegenerative diseases, including Alzheimer’s disease, Parkinson’s disease, or amyotrophic lateral sclerosis, on glioma risk. Nonetheless, an epidemiological study described inverse associations between glioma risk and Alzheimer’s disease risk and a link between glioma risk and increased risk of multiple sclerosis have been described.^84^ Similarly, while most psychiatric traits do not show a clear relationship, genetical variants associated with schizophrenia has been associated with increased odds of non-GBM glioma.^85^ Additionally, while the impact of chronic stress on GBM aggressiveness has also been investigated, conflicting results preclude a clear association.^86^^,^^87^ Taken together, these findings suggest that, although particular associations may exist, current evidence does not support a broad role of neurological diseases in driving glioma development. Nonetheless, it is important to note that a pathologically altered brain, as observed in neurological and psychiatric conditions, may present distinct signaling landscapes and altered cellular interactions that could potentially shape neuron-glioma crosstalk in ways that remain largely unexplored. In contrast, the impact of glioma on neural function, including the induction of hyperexcitability and seizures, is well established.

To overcome all these multifaceted challenges, interdisciplinary collaboration and data integration are essential. Combining spatial, molecular, imaging, and functional insights will be critical to exploit the already known and new interactions between tumor cells and neurons in the context of brain tumors, which might further be integrated in the clinical context.

## 6 Conclusions

In conclusion, complex interactions between malignant gliomas and normal brain cells, particularly neurons, have emerged as critical drivers of tumor aggressiveness. These bidirectional connections fuel tumor progression and ultimately impact the clinical course by aggravating neurological symptoms and reducing patient survival. While this field is still being explored, emerging data suggests that disrupting neuron-glioma crosstalk may hold therapeutic potential for these highly aggressive brain tumors. A deeper and more integrative understanding of the brain-tumor connectome, together with refined molecular profiling (e.g., spatially-resolved and/or single-cell data), may enable better patient stratification and guide precise, targeted treatment strategies.

## CRediT authorship contribution statement

**Eduarda P. Martins:** Conceptualization, Investigation, Writing – original draft, Writing – review & editing. **Helena M. Figueiredo:** Conceptualization, Investigation, Writing – original draft, Writing – review & editing. **Nuno Sousa:** Conceptualization, Supervision, Writing – review & editing. **Bruno M. Costa:** Conceptualization, Supervision, Writing – review & editing.

## Funding

This work was supported by projects and grants from FCT – Foundation for Science and Technology (grant number: 2022.04859), Foundation for Science and Technology (grant numbers: UID/06304/2025 and LA/P/0050/2020), and Norte Portugal Regional Operational Programme (grant number: NORTE2030-FEDER-02705300).

## Declaration of generative AI and AI-assisted technologies in the writing process

During the preparation of this work, the authors used ChatGPT (OpenAI) in order to improve readability and language. After using this tool, the authors reviewed and edited the content as needed and take full responsibility for the content of the published article.

## Declaration of competing interest

The authors declare that they have no known competing financial interests or personal relationships that could have appeared to influence the work reported in this paper.
